# New Appliances and Things Medical

**Published:** 1893-07-15

**Authors:** 


### NEW APPLIANCES AND THINGS MEDICAL.

[All preparations, appliances, novelties, etc., of which a notice is desired, should be sent for the Editor, to care of The Manager, 428, Strand, London, W.C.]

### TABELLÆ.

Messrs. Allen and Hanbury's speciality in the preparation of compressed drugs is a marvel of pharmaceutical enterprise. In appearance their tabellæ hardly differ from the preparations of compressed drugs that this firm and several others have made so familiar to the profession and the public. There is no doubt, however, that this neatness and compactness was brought about by a very high compression, which had the drawback of rendering some of the more insoluble drugs so difficult of disintegration that one could not always rely on the absorption of the drug exhibited. Messrs. Allen and Hanbury, recognising this distinct disadvantage, set to work to overcome the difficulty, and we congratulate them on the entire success of their efforts. We have used their preparations extensively, and have been very pleased with the results, but were inclined to think that their claim that the firm's hard tabellæ would disintegrate within five minutes must be fanciful, till experiment proved that even the insoluble bismuth and sulphur tabellæ completely disintegrated in cold water in twenty seconds. The most recently introduced drugs will be found included in their list, including the thyroid gland, which thus is made most acceptable to the patient.

### MESSRS. HOCKIN, WILSON, AND CO.'S PREPARATIONS.

This reputed firm have brought under our notice several pharmaceutical preparations that deserve the attention of practitioners who recognise the valuable aid to practice afforded by high-class pharmacy. The Liquor Podophyllin is a soluble preparation freely miscible with water, acids, alkalies, decoctions, æthers, and tinctures, and will be found one of the most palatable and efficient means of exhibiting this drug. Their Liq. Podophyllin et Bellad. c Strychn. and Liq. Pod. et Pepsin are useful combinations. The albuminate of iron has long been a favourite with us, and it has the advantage over the inorganic compounds of iron of being directly assimilated by the blood. The amount of albuminate that need be taken to get all the beneficial results of a course of iron is very much smaller than is the case with the more commonly-used salts of iron, and consequently the digestive tract, however delicate, is not disturbed. The Extract Salix Nigra Liq. will be found to give relief in ovarian pain to a variety of causes. We may congratulate Messrs. Hockin on the Ung. Antiseptici Adhesivum, of which we have received a sample. It is most cleanly in appearance, and while it is quickly removed by warm water, it will be found a very efficient means for applying dressings to parts where it is difficult to apply ordinary dressings.

### MILK OF MAGNESIA.

This is a palatable fluid preparation of magnesia, which will be most acceptable to children, for whom it is particularly adapted. It is brought out by Messrs. Chas. H. Phillips, of New York, from whom we have also received samples of a combination of quinine with "wheat phosphates" and of their palatable cod liver oil emulsion, both of which are a credit to the firm.

### LEVICO WATER.

The Levico-Natural Arsenio-Ferric Water is one of the most reliable and potent means of administering iron and arsenic in natural solution. It has stood the test of clinical experience, and is now extensively prescribed for many forms of anæmia and in skin diseases where arsenic is indicated. It is sent out in two strengths, the mild and the strong, from the two distinct springs.

### ROSBACH WATER.

This natural sparkling table water is bottled direct from the spring in Germany, and is, without exception, the most palatable natural water we know. It contains a very small proportion of earthy salts, and for this reason is well suited for habitual and daily use, and presents many advantages over some of the better known sparkling table waters. We have found that even with the finest vintages of wine the original flavour is but little impaired by this bright and delicious water. It is undoubtedly the perfection of table waters.

